# Inhospital Exercise Training in Children With Cancer: Does It Work for All?

**DOI:** 10.3389/fped.2018.00404

**Published:** 2018-12-19

**Authors:** Javier S. Morales, Julio R. Padilla, Pedro L. Valenzuela, Elena Santana-Sosa, Cecilia Rincón-Castanedo, Alejandro Santos-Lozano, Alba M. Herrera-Olivares, Luis Madero, Alejandro F. San Juan, Carmen Fiuza-Luces, Alejandro Lucia

**Affiliations:** ^1^Faculty of Sport Sciences, Universidad Europea de Madrid, Madrid, Spain; ^2^Systems Biology Department, University of Alcalá, Alcalá de Henares, Madrid, Spain; ^3^i+HeALTH Research Group, Department of Health Sciences, Miguel de Cervantes European University, Valladolid, Spain; ^4^Instituto de Investigación Sanitaria Hospital 12 de Octubre (“i+12”), Madrid, Spain; ^5^Hospital Universitario Infantil Niño Jesús, Madrid, Spain; ^6^Departamento de Salud y Rendimiento Humano, Universidad Politécnica de Madrid, Madrid, Spain

**Keywords:** fitness, pediatric cancer, functional mobility, physical activity, solid tumors, exercise is medicine

## Abstract

**Purpose:** Physical exercise training might counteract the weakening effects of both pediatric cancer and anti-cancer treatment. We aimed to analyze the prevalence of “responders” and “non-responders” to inhospital exercise training in children with cancer and to identify the factors that could influence responsiveness, which might help personalize exercise interventions for this patient population.

**Methods:** We performed an ancillary analysis of the randomized controlled trial “Physical activity in Pediatric Cancer” (NCT01645436), in which 49 children with solid tumors were allocated to an inhospital exercise intervention or control group. The present study focused on the children in the former group (*n* = 24, 10 ± 4 years), who performed 3 weekly training sessions (aerobic + strength exercises). The intervention lasted 19 ± 8 weeks (i.e., from the start to the end of neoadjuvant chemotherapy treatment). A responder-vs-non-responder analysis was performed for physical capacity-related endpoints (five-repetition maximum strength, functional mobility tests, and cardiorespiratory fitness [CRF]). Only those participants showing improvements in a given test of a magnitude greater than both the random error and the threshold for clinically meaningful changes were considered responders.

**Results:** Most participants improved their performance in the strength tests, with 80, 88, and 93% of total showing a positive response for seated bench press, lateral row, and leg press, respectively (*p* < 0.001). No significant improvements were observed for the functional mobility tests or CRF (*p* > 0.05, rate of responsiveness ≤ 50%). No differences between responders and non-responders were observed for sex, age, type of cancer, or treatment (i.e., including or not anthracyclines/radiotherapy). However, significant differences (*p* < 0.05) were observed between responders and non-responders for baseline performance in all the tests, and a significant (*p* < 0.05) inverse relationship was found between baseline performance and relative improvement for most endpoints.

**Conclusions:** Although most children improved their muscle strength after the exercise intervention, a considerable individual variability was observed for the training responsiveness of functional mobility and CRF. A lower baseline performance was associated with a higher responsiveness for all the study endpoints, with the fittest children at the start of treatment showing the lowest responses. Efforts to individualize exercise prescription are needed to maximize responsiveness in pediatric cancer patients.

## Introduction

With advances in cancer treatment, nowadays almost 80% of children diagnosed with cancer will survive the disease (1). However, anti-cancer therapies and the disease itself are associated with a deterioration of patients' physical fitness (2), as reflected by low levels of cardiorespiratory fitness (CRF) and muscle strength that are present both during (3, 4) and after the treatment (3, 5). Children with cancer also have a decreased ability to perform activities of daily living (6), which negatively affects their well-being and health-related quality of life (HRQoL) (7).

Although the impaired physical fitness of pediatric cancer patients is likely multifactorial, a main contributor is their typically reduced activity levels (4). In this context, meta-analytical evidence shows positive effects of exercise intervention on the CRF, muscle strength, functional mobility, HRQoL, and daily physical activity of children with cancer (8, 9). However, the great majority of studies in the field have been performed in children with acute lymphoblastic leukemia (10–18) and the evidence for the effects of exercise training interventions in children with other types of cancer is much scarcer (19–22). On the other hand, exercise benefits are typically reported under the assumption that the group average represents the response of most individuals. Yet, a wide inter-individual variability can be observed in the human response to a given training program, which results in subjects being classified as responders (i.e., those who achieve clinically meaningful benefits) or non-responders (i.e., those who experience a worsening or remain unchanged) (23, 24). Such individual variability has been reported in healthy people or in individuals with different disease conditions (25, 26) but not in cancer patients. Thus, the aim of this study was to analyze the prevalence of responders/non-responders to an inhospital exercise (aerobic + strength) training program for physical fitness and functional mobility in children with solid tumors, as well as to identify the factors that could influence individual responsiveness.

## Materials and Methods

### Participants and Study Design

We performed an ancillary analysis of the randomized controlled trial “Physical activity in Pediatric Cancer” (NCT01645436), in which 49 children with solid tumors were randomly allocated to an inhospital exercise intervention or control group. The present study focused on the former group, with patients performing 3 weekly training sessions (aerobic + strength exercises) during 19 ± 8 weeks on average (i.e., from the start to the end of neoadjuvant chemotherapy treatment). All participants, together with their parents/guardians, gave their written informed consent, and the study was approved by the institutional Ethics Committee.

### Exercise Intervention

Participants followed an inhospital training program, which took place during the entire neoadjuvant chemotherapy treatment period. The program has been previously described in detail (21). Briefly, the exercise intervention included three sessions per week (Monday-Wednesday-Friday), each lasting ~60–70 min. Each session included a pre-conditioning period of ~30 min of aerobic exercise (cycle-ergometer pedaling, treadmill running, or arm cranking in those children missing a lower limb due to the disease, and aerobic games). The training load was gradually increased depending on the age, physical capacity and health status of each child. Exercise intensity was recorded continuously with heart rate (HR) monitors and corresponded to 60–70% of the maximum HR value determined during the baseline tests for CRF assessment (see below). Aerobic exercise was followed by ~30 min of strength training. The latter took place in the hospital gymnasium, which is appropriately equipped for this purpose (27) or in the ward room (especially during neutropenic episodes). Two to three sets (8–15 repetitions with a rest period of 1–2 min between sets) of the following exercises were performed in each session: shoulder, chest and leg presses, side-arm rowing extension and flexion, knee extension and flexion, and abdominal, lumbar, and shoulder adduction. The load was gradually increased as the strength of each child improved (i.e., by ~2 kg after three training sessions with a given weight) and independently for each exercise, starting at 50% of the baseline five-repetition maximum (5RM). In the ward sessions, dumbbell exercises were similar to those performed in the gymnasium with weight training machines.

Before each training session, we enquired the medical staff about the children's health status in order to determine if they could exercise that day. No session started without medical permission. Depending on the clinician's recommendations, missing sessions were performed on another week day (Tuesday or Thursday).

### Endpoints

Endpoints were assessed at baseline (i.e., upon initiation of neoadjuvant chemotherapy) and upon treatment termination (i.e., 19 ± 8 weeks later). Baseline assessment was preceded by three familiarization sessions with each test. The analyzed endpoints have been previously described in detail (21, 27). Briefly, muscle strength was assessed in the hospital gymnasium with pediatric-specific weight training machines (Strive, Inc., McMurray, PA) (21, 27). We evaluated the 5RM for leg press, seated bench press and seated lateral row as previously described (28). Each subject was instructed to perform all the exercises to momentary muscular exhaustion. Any repetition not performed with a full range of motion was not counted. Children with an amputated lower limb did not perform the leg press test.

Functional mobility was assessed with the 3-meter Timed Up and Go (TUG) and Timed Up and Down Stairs (TUDS) tests (27). Performance time was measured by the same investigator with the same stopwatch to the nearest 0.1 s.

CRF (peak oxygen uptake, VO_2peak_) was determined using “breath-by-breath” analysis with a metabolic cart (Vmax 29C; SensorMedics Corp., Yorba Linda, CA) and pediatric masks during a ramp-like treadmill testing protocol (Technogym Run Race 1400HC; Cesena, Italy) as previously described (27). For those children missing a lower limb and who were thus unable to perform the treadmill testing, we performed the test with an arm crank ergometer (Monark Rehab Trainer model 881E; Varberg, Sweden).

### Responsiveness Analysis

Responsiveness was defined as positive changes whose magnitude exceeded both the random error (which includes the technical error of biological measurements and the day-to-day biological variability) and the expected threshold for clinically meaningful benefits.

Random error was defined as two times the typical error (TE) of measurement (29). The TE was calculated for each test as the standard error of within-subject standard deviation (SD) (30), obtained from previous reliability tests performed in the same hospital and with the same equipment by children with hematological cancer for leg press, bench press, lateral row TUGS, and TUDS tests (12). Participants performed each test twice separated by 48 h, and intra-class correlation coefficients and random error were calculated, yielding the following values: correlation coefficient of 1.00, 1.00, 1.00, 1.00, and 0.99 for leg press, bench press, lateral row, TUDS, and TUG, respectively; and random error of 7.3 kg, 3.2 kg, 4.5 kg, 0.3 s and 0.2 s, respectively (12). No reliability analysis (and consequently no random error) was available for CRF. Moreover, as no information was found in the scientific literature regarding the biological threshold of clinically meaningful changes for the tests and patient population of our study, one-fifth of the between-subject SD at baseline was taken as the as threshold for clinically relevant improvements as proposed by Hopkins et al. (31) and Hecksteden et al. (32).

The thresholds for clinically meaningful changes for leg press, bench press, lateral row, TUDS, TUG, and CRF were 5.0 kg, 3.7 kg, 3.7 kg, 1 s, 0.2 s, and 1.6 ml/kg/min, respectively. Only those children showing improvements in a given test of a magnitude greater than both the random error and the threshold for clinically meaningful changes were considered responders. A responder-vs.-non-responder analysis was performed only when more than 25% of participants were considered non-responders for a given endpoint.

### Statistical Analysis

Data are presented as mean ± SD unless otherwise stated. The normal distribution (Shapiro-Wilk test) and homoscedasticity (Levene's test) of the data were checked before any statistical treatment.

Differences between mean values of baseline and post-intervention data were assessed using Student's paired *t-*tests. Differences between responders and non-responders were assessed using the Mann-Whitney *U* test for continuous variables (age, Tanner stage of maturation, training sessions, and baseline performance) and the Fishers' exact test for proportions (sex, type of solid tumor, treatment or not with radiotherapy or anthracyclines).

The relationship between the different variables and the likelihood of being a responder was assessed using univariate logistic regression analyses, whereas the relationship between baseline performance and relative improvement was assessed using Pearson's correlation analysis. Correlation coefficients (*r*) values of 0.1, 0.3, 0.5, 0.7, and 0.9 were considered small, moderate, strong, very strong, and extremely strong, respectively (33). All statistical analyses were conducted using a statistical software package (SPSS 23.0, USA) setting the significance level at 0.05.

## Results

The mean age of the studied population was 10 ± 4 years, ranging from 4 to 16 years (Table 1). According to the International Classification of Childhood Cancer (34), five main diagnostic groups of cancer were included (i.e., lymphomas and reticuloendothelial neoplasms; soft tissue and other extraosseous sarcomas; malignant bone tumors; neuroblastoma and other peripheral nervous cell tumors; and germ cell tumors, trophoblastic tumors and neoplasms of gonads). The duration of the intervention was 19 ± 8 weeks, ranging from 9 to 41 weeks, and participants performed 35 ± 14 training sessions on average. Mean adherence to the intervention was 63% ± 21%. No major adverse events or health-related issues attributable to the testing or training sessions were noted.

**Table 1:** Main demographic and clinical characteristics of the study participants at baseline (i.e., upon diagnosis).

	***n* = 24**
Male (%)	17 (71%)
Age (years)	10 ± 4
**TANNER MATURATION STAGE**	
I	42%
II	4%
III	21%
IV	8%
V	25%
TYPE OF TUMORS^*^	
**Lymphomas and reticuloendothelial neoplasms**	
Hodgkin lymphoma	4%
Non-Hodgkin lymphomas	34%
**Soft tissue and other extraosseous sarcomas**	
Rhabdomyosarcoma	8%
Other specified soft tissue sarcomas	
*Non-rhabdomyosarcoma (synovial sarcoma)*	4%
**Malignant bone tumors**	
*Ewing's Sarcoma*	25%
*Osteosarcoma*	13%
**Neuroblastoma and other peripheral nervous cell tumors**	
Neuroblastoma	4%
**Germ cell tumors, trophoblastic tumors, and neoplasms of gonads**	
Malignant gonadal germ cell tumors	
*Germinomas*	4%
*Non-germinomas (embryonic carcinoma)*	4%
**Main treatment characteristics**	
Total duration (weeks)	19 ± 8
Chemotherapy cycles (number)	7 ± 3
In-room isolation episodes due to neutropenia (number)	3 ± 2
**Anthropometric variables**	
Body mass (kg)	42.6 ± 19.9
BMI (kg/m^2^)	19.2 ± 5.0

*Data are mean ± SD*.

* *Type of tumors were classified according to the International Classification for Childhood Cancer. BMI, body mass index*.

A significant improvement in mean values was observed after the exercise intervention compared to baseline for all the strength tests (*p* < 0.001, rate of responsiveness >80%), but not for functional mobility tests or CRF (*p* > 0.05) (Table 2). However, the individual responsiveness analysis revealed that 31–53% of participants showed a meaningful improvement in TUG, TUDS, and CRF (Tables 2, 3).

**Table 2:** Effects of an inhospital exercise intervention on study endpoints.

**Test**	***N* with data**	**Baseline**	**Post-intervention**	**Change (SE)**	***p*-value**	**β (95% CI)**	**Responders N (%)**
Seated bench press (kg)	20	30 ± 18	40 ± 20	11(1.9)	< 0.001	0.6(0.4, 0.8)	16(80%)
Seated lateral row (kg)	17	29 ± 19	43 ± 21	15(2.1)	< 0.001	0.8(0.6, 1.0)	15(88%)
Leg press (kg)	14	35 ± 25	67 ± 35	32(6.2)	< 0.001	1.3(0.8, 1.8)	13(93%)
TUG (s)	15	4.0 ± 1.1	3.8 ± 0.5	−0.3(0.2)^*^	0.125	−0.3(−0.6, 0.1)	8(53%)
TUDS (s)	13	8.9 ± 5.0	7.0 ± 1.0	−1.9(1.3)^*^	0.159	−0.4(−0.9, −0.1)	4(31%)
CRF (ml/kg/min)	21	25.0 ± 8.0	25.0 ± 5.2	0(1.3)	0.960	0.0(−0.3, −0.3)	8(38%)

Data are mean ± SD. P-values were determined using student's paired t tests. β corresponds to the change expressed in standardized units (i.e., divided by baseline SD). Data for the strength tests correspond to the 5-repetition maximum. Changes are expressed as mean delta change along with the standard error (SE). Responsiveness was determined as a positive change greater than both the typical error of measurement and the minimal clinically meaningful change. CRF, cardiorespiratory fitness; ES, effect size; TUDS, timed up and down stairs test; TUG, timed up-and-go 3-meter test; VO_2peak_, peak oxygen uptake. Symbol:

* *a negative value presents an actual performance improvement in the test*.

**Table 3:** Individual response to the different performance tests.

**Subject**	**Seated bench press**	**Seated lateral row**	**Leg press**	**TUG**	**TUDS**	**CRF**
1		N/A	+		N/A	+
2	+	N/A	+	+	N/A	+
3	+	+	+			+
4		+	+			+
5	+	+	+	+	+	+
6	+	+	N/A	N/A	N/A	
7		+	N/A	N/A	N/A	
8	N/A	N/A	+	N/A	N/A	N/A
9	+			+	+	
10	+	N/A	N/A	N/A	N/A	+
11		+	N/A			
12	+	+	+	+	+	+
13	+	+	N/A	N/A	N/A	+
14	+	+	N/A	N/A	N/A	+
15	+	+	+	+	+	
16	+	+	+			
17	+	+	N/A	N/A	N/A	N/A
18	+	+	+			+
19	+	N/A	+		+	
20	N/A	N/A	N/A	N/A	N/A	
21	+	+	+		+	+
22	+	+	N/A			
23	N/A	N/A	+		+	
24		N/A	N/A	N/A	N/A	N/A

*Responsiveness (indicated as “+”) was determined as a positive change greater than both the typical error of measurement and the minimal clinically meaningful change. CRF, cardiorespiratory fitness; N/A, not available; TUDS, timed up and down stairs test; TUG, timed up-and-go 3-meter test*.

**Figure 1:**
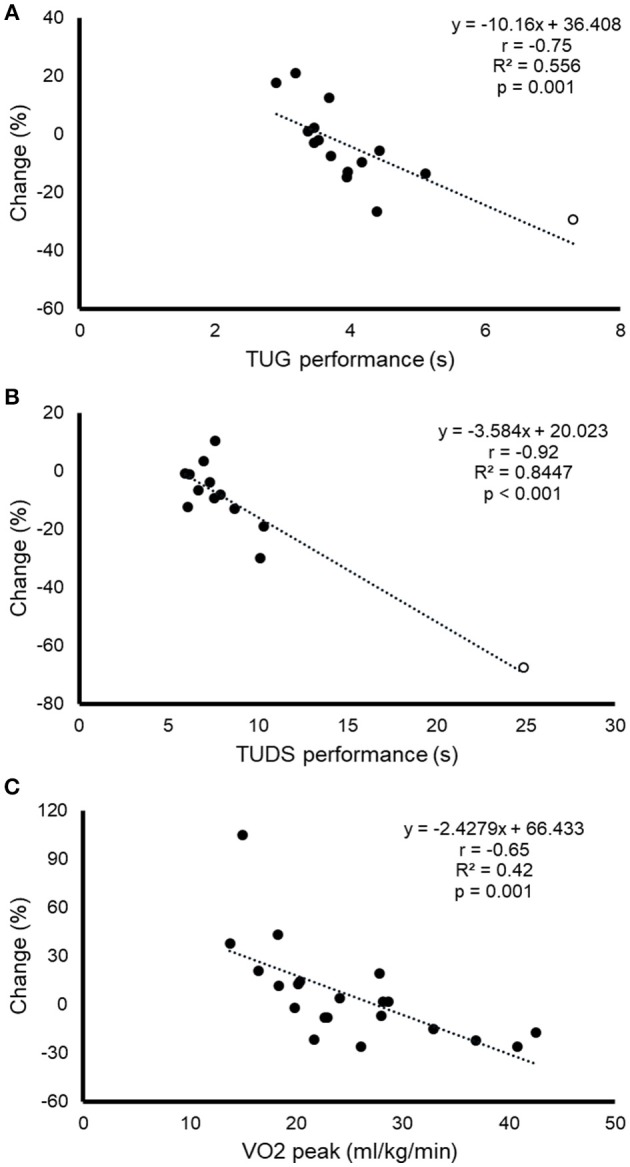
Relationship between baseline performance in the timed up and go (TUG) test **(A)**, the timed up and down stair (TUDS) test **(B)** and cardiorespiratory fitness [CRF, expressed as peak oxygen uptake [VO_2peak_]), **(C)**], on the one hand, and the relative change in performance for each test after the exercise training intervention, on the other. When outliers [white dots in **(A,B)**] were removed from the analysis, the relationship was still significant for both TUG (*r* = −0.74, *p* = 0.002) and TUDS (*r* = −0.68, *p* = 0.01).

**Figure 2:**
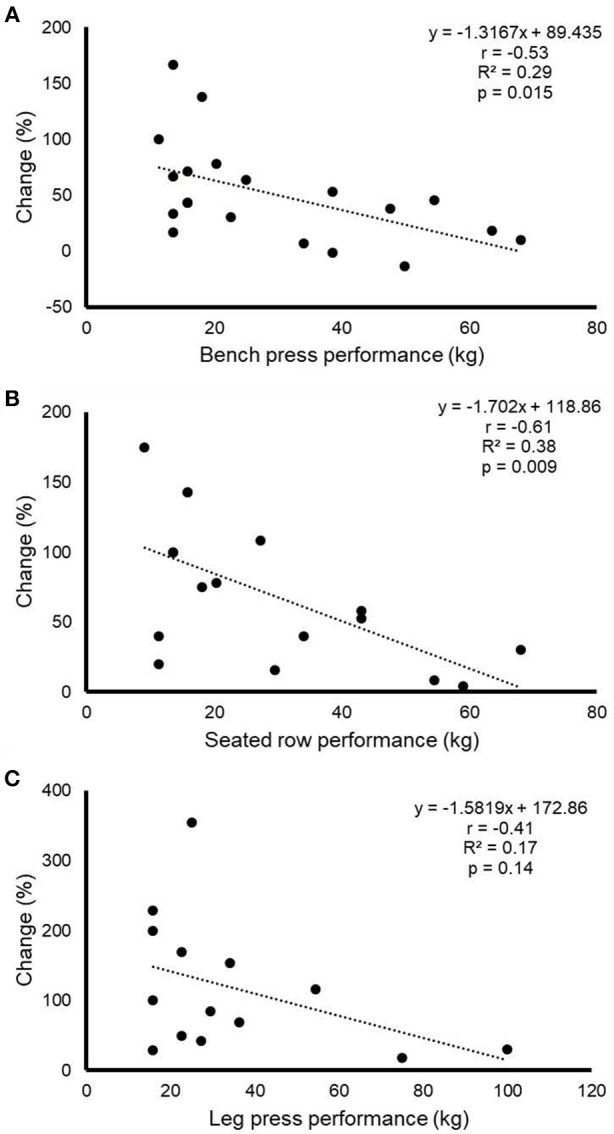
Relationship between baseline performance in bench press **(A)**, seated row **(B)** and leg press **(C)**, on the one hand, and the relative change for performance in each test after the exercise training intervention, on the other.

The results of the responder-vs.-non-responder analysis for functional mobility tests and CRF are shown in Table 4. No significant differences were observed between responders and non-responders for sex, age, biological maturity (Tanner stage), cancer type, or treatment (i.e., anthracyclines or radiotherapy) (Table 4). Significant differences in baseline performance were observed between responders and non-responders for all the tests (*p* < 0.05). Moreover, a significant and strong inverse relationships (*p* < 0.05) was found between baseline performance and relative improvement for both functional mobility tests and CRF (Figure 1). A significant inverse relationship between baseline and relative improvement was also observed for bench press and lateral row strength tests (Figure 2). Finally, a significant relationship between baseline performance and the likelihood of being responder was found for CRF (OR: 0.656, 95%CI: 0.449–0.958; *p* < 0.05), but no other descriptive value was found to increase the likelihood of being responder (*p* > 0.05 for all).

**Table 4:** Differences between responders (R) and non-responders (NR).

	**TUG**			**TUDS**			**CRF**		
	**R (*n* = 8)**	**NR (*n* = 7)**	***p*-value**	**R (*n* = 4)**	**NR (*n* = 9)**	***p*-value**	**R (*n* = 8)**	**NR (*n* = 13)**	***p*-value**
Male (%)	5 (62%)	7 (100%)	0.200	3 (75%)	8 (89%)	1.000	5 (63%)	10 (77%)	0.477
Age (years)	9 (6)	13 (7)	0.072	9 (6)	12 (6)	0.148	9 (6)	12 (6)	0.210
Tanner (stage)	1 (2)	3 (4)	0.152	1 (2)	3 (4)	0.148	1 (2)	3 (4)	0.185
Cancer type (% lymphoma)	4 (50%)	4 (57%)	1.000	2 (50%)	5 (56%)	1.000	3 (38%)	5 (38%)	1.000
Treatment with anthracyclines (%)	7 (87%)	5 (71%)	0.569	4 (100%)	7 (78%)	1.000	8 (100%)	11 (85%)	0.505
Anthracycline dose >300 mg/m^2^ (%)	2 (25%)	1 (14%)	1.000	1 (25%)	2 (22%)	1.000	6 (75%)	4 (31%)	0.080
Treatment with radiotherapy (%)	4 (50%)	2 (29%)	0.608	2 (50%)	3 (33%)	1.000	3 (38%)	6 (46%)	1.000
Training sessions (n)	28 (24)	36 (27)	0.779	28 (32)	26 (26)	0.604	36 (15)	28 (28)	0.595
Baseline performance^*^	4.3 (1.0)	3.5 (0.3)	**<0.001**	10.2 (12.2)	7.0 (1.5)	**0.003**	18 (5)	28 (12)	**0.001**

*Data are shown as median and interquartile range (continuous variables) or as number and percentage (dichotomous variables)*.

* *Baseline performance is expressed in seconds for TUG and TUDS, and in ml/kg/min (peak oxygen uptake) for CRF. Differences were calculated using the Mann-Whitney U (continuous variables) and Fisher's exact test (proportions). The odds ratio (OR), calculated using univariate logistic regression, represents the likelihood of being responder attending to that specific variable. CRF, cardiorespiratory fitness; ES, effect size; N/A, not available; NR, non-responders; R, responders; TUDS, timed up and down stairs test; TUG, timed up-and-go 3-meter test. Significant P-values are in bold*.

## Discussion

The results of this ancillary analysis show that an inhospital exercise program can be safely applied to increase muscle strength in pediatric cancer patients with solid tumors undergoing neoadjuvant treatment, with a high prevalence of responders (i.e., >80%). The rate of responsiveness, however, was considerably lower for CRF or functional mobility tests (i.e., half and one third of the participants showed a meaningful improvement in the TUG test/CRF and in the TUDS test, respectively). On the other hand, significant differences were observed between responders and non-responders in baseline physical performance for all the tests: that is, non-responders had a better baseline performance.

A significant inverse relationship was found between baseline performance and relative performance improvement in both functional mobility tests, CRF, and in two of the three strength tests (bench press, lateral row), suggesting that a greater training stimulus might be needed in the fittest children. In fact, previous research has also found non-responders to training among children. For instance, ~25% of children with insulin resistance who performed a short-duration (6 weeks) resistance training intervention showed a negative response for muscle strength (35). Others have reported a prevalence of non-responders for CRF of ~20% among healthy young individuals (36).

Previous evidence shows that some exercise variables, such as training frequency and intensity, can be manipulated to enhance responsiveness. For instance, a systematic review concluded that intense aerobic exercise training elicits greater improvements in fitness and cardiometabolic risk markers than moderate-intensity aerobic programs (37). Montero and Lundby (38) recently reported that the prevalence of non-responders for CRF after a 6 week endurance training program progressively declines with training duration (from 60 min per week to longer durations), with all the participants responding positively when exercising >240 min per week. Similarly, Ross et al. (39) found that, for a given exercise intensity, increasing exercise volume reduced the rate of non-responders by 50%, whereas increasing exercise intensity (from 50 to 75% of CRF) for a fixed exercise volume fully avoided non-responsiveness. Thus, an individual initially classified as non-responder to a certain training stimulus might actually respond to a different type of training program (40) or to a higher training volume (38, 39) or intensity (39). The children in our study who were non-responders had a poor baseline CRF (mean VO_2peak_ of 29 ml/kg/min) compared to children without a previous history of cancer of similar age, gender and sexual maturity (i.e., mean VO_2peak_ of 46 ml/kg/min) (41). Considering the importance of CRF as one of the strongest indicators of health status (42), efforts to enhance responsiveness in these patients are needed, which might probably involve applying a higher training stimulus (that is, higher intensity and/or volume) (24).

Our study has some limitations, including mainly heterogeneity in several participants' characteristics (i.e., type of solid tumor, age, or sexual maturity) and the small sample size. However, heterogeneity in variables such as sexual maturity allowed us to account for the influence of maturation status on individual responsiveness, with the more sexually mature participants requiring a higher training stimulus. Further research with larger cohorts might allow for the examination of responsiveness predictors or for a prediction model. Concerning the low sample size, it should be noted that childhood cancer is a rare disease with pediatric solid tumors being particularly unusual (43), which implies an enormous recruitment challenge. In fact, it took more than 3 years to complete the participants' recruitment for this study (19–22). On the other hand, not all the participants could perform all the pre- and post-intervention tests. Another limitation was that no reliability analysis (and consequently no TE) was available for VO_2peak_, and thus in the case of CRF responsiveness was solely determined attending to the minimal clinically meaningful change. Finally, the relationship between baseline performance and the observed improvement could be partly due to the learning of the technique (i.e., in those patients with the lowest baseline performance) and to statistical artifacts, especially for those subjects whose results are particularly far from the mean (whether too high or too low) (44). In this regard, we performed three familiarization sessions, and analyses were performed using percentage relative changes to minimize the influence of the “regression to the mean” phenomenon. In turn, major strengths of our study are the novelty of our approach and the clinical relevance of the topic.

## Conclusions

The inclusion of inhospital exercise interventions in children with solid tumors undergoing neoadjuvant treatment improved muscle strength safely. However, a considerable individual variability was observed for the improvements in functional mobility and CRF. A lower baseline performance was associated with a better responsiveness for most tests, with those children with the best physical status at the start of treatment showing the lowest responses. Thus, our results might be taken into account in future efforts to prescribe effective, personalized exercise programs in pediatric cancer patients. Future research might determine if applying a higher training stimulus (i.e., higher intensity and/or volume) might maximize responsiveness in these patients.

## Ethics Statement

This study was carried out in accordance with the recommendations of the Research Ethics Committee of the Children's Hospital Niño Jesús (Madrid, Spain). The protocol was approved by the Research Ethics Committee of the Children's Hospital Niño Jesús (Madrid, Spain; approval number R-0007/13). All subjects gave written informed consent in accordance with the Declaration of Helsinki.

## Author Contributions

AL, CF-L, JM, and PV conceived and designed the study. AH-O, AS, CR-C, ES-S, and JP supervised the training sessions and performed the evaluations. AS, AS-L, JM, and PV analyzed the data. AL, JM, and PV drafted the manuscript. AL, CF-L, and LM edited the final manuscript. All authors reviewed and approved the final version of the manuscript.

### Conflict of Interest Statement

The authors declare that the research was conducted in the absence of any commercial or financial relationships that could be construed as a potential conflict of interest.
